# Classification of Multiclass Histopathological Breast Images Using Residual Deep Learning

**DOI:** 10.1155/2022/9086060

**Published:** 2022-10-10

**Authors:** Mohamed Meselhy Eltoukhy, Khalid M. Hosny, Mohamed A. Kassem

**Affiliations:** ^1^Department of Information Technology, College of Computing and Information Technology at Khulais, University of Jeddah, Jeddah 21959, Saudi Arabia; ^2^Department of Information Technology, Faculty of Computers and Informatics, Zagazig University, Zagazig 44519, Egypt; ^3^Department of Robotics and Intelligent Machines, Faculty of Artificial Intelligence, Kafrelsheikh University, Kafr El-Shaikh 33511, Egypt

## Abstract

Pathologists need a lot of clinical experience and time to do the histopathological investigation. AI may play a significant role in supporting pathologists and resulting in more accurate and efficient histopathological diagnoses. Breast cancer is one of the most diagnosed cancers in women worldwide. Breast cancer may be detected and diagnosed using imaging methods such as histopathological images. Since various tissues make up the breast, there is a wide range of textural intensity, making abnormality detection difficult. As a result, there is an urgent need to improve computer-assisted systems (CAD) that can serve as a second opinion for radiologists when they use medical images. A self-training learning method employing deep learning neural network with residual learning is proposed to overcome the issue of needing a large number of labeled images to train deep learning models in breast cancer histopathology image classification. The suggested model is built from scratch and trained.

## 1. Introduction

The most common diagnosed cause of death from cancer in women is breast cancer. Every year about 2.1 million women have breast cancer worldwide, according to the World Health Organization (WHO) [1, 2]. Four forms of breast tissue are present, i.e., normal, benign, in-situ carcinoma, and invasive carcinoma. Benign tissue leads to small structural changes in the breast but is not known as cancer and is not harmful to health in most circumstances. The malignant tumor usually spreads by another organ, which is called invasive carcinoma. In-situ carcinoma is in the lobule system of the mammalian canal and has little effect on any organ. In-situ carcinoma can be treated if it is diagnosed in time.

Many methods have been presented for breast cancer detection [3–7]. In addition, there are many procedures and modalities for the diagnosis of breast cancer, such as 3-D Ultrasound (US), X-ray mammography, Positron Emission Tomography (PET), Computed Tomography (CT), breast temperature measurement, and Magnetic Resonance Imaging (MRI). Pathological diagnoses are sometimes considered the “golden standard.” To be properly observed and analyzed, removed tissue can normally be stained where the most appropriate approach is the Hematoxylin and Eosin. The hematoxylin teases dark purple nuclei, and other structures (cytoplasm, stroma, etc.) are pink.

Artificial Intelligence (AI) technology has been growing rapidly in recent years. Especially, it makes significant progress in image processing, recognition, analysis, and computer vision [8]. In histopathological research, AI also perceived potential advantages. Diagnosis with AI can carry out tedious focus tests and easily collect useful diagnostic results from huge data. In the meantime, AI has a high quantitative analytical capability for histopathological diagnosis and can prevent manual analysis-based subjective discrepancies. Pathologists may reduce their misjudgment and increase their work performance in certain ways.

In medical image analysis, deep learning algorithms have been applied by several researchers [9–11]. Deep learning's performance is largely dependent on the availability of many training samples from which to learn the descriptive feature mappings of the images, resulting in extremely accurate classification results [12–15].

Some researchers use breast-density scores as a sign of early detection of breast cancer. Kallenberg et al. [16] and Dhungel et al. [17] used the breast-density estimation score method for the early detection of breast cancer. Many deep learning approaches can be found in the literature that uses transfer learning, e.g., [18, 19], to utilize the image features to classify normal and abnormal regions.

Researchers in [20] use multistage fine-tuned CNN by utilizing multistage transfer learning for multi-view regions of interest (ROIs) to classify the masses into malignant and benign classes. Another study [21] proposed a hybrid multistage fully convolutional network (FCN) be combined with conditional random field (CRF) for the detection of masses in multi-view mammogram images.

Breast histopathology analysis is the gold standard for diagnosing breast cancer. Deep learning-based classification methods using breast histology images have made the analysis process simple and fast in recent years. The first deep learning approach using the BreaKHis was originally published by Spanhol et al. [22]. Bayramoglu et al. [23] proposed a deep learning method to classify histopathology images without considering the magnification factors. Another deep learning multi-classification model was proposed by Han et al. [24]. The proposed class structure-based Deep Convolutional Neural Network (CSDCNN) as an end-to-end 51 recognition method adopts a hierarchical representation with feature space constraints that maximize the Euclidean distance of interclass labels. A CNN-based solution is also presented by Das et al. [25]. They employ pretrained CNN transfer learning for specific breast histopathology features and majority voting using random multiviews at multi-magnification. Zhi et al. [26] proposed an ensemble model containing three custom Convolutional Neural Network (CNN) classifiers using transfer learning. Motlagh et al. [27] have examined different Inception models and ResNet architectures on digital cancer images, including BreaKHis. Aiming to prepare the data for further feature extraction and classification, the authors have applied color map selection and data augmentation as preprocessing steps. Wang et al. [28] proposed a dependency-based lightweight convolutional neural network (DBLCNN) for the multi-classification task of breast histopathology images.

To generate and choose pseudo-labeled samples for categorizing breast cancer histopathology images, Asare et al. [29] integrated self-training and self-paced learning. For the multiclass classification of breast digital pathological images (normal tissue, benign lesion, ductal carcinoma in-situ, and invasive cancer), Mi et al. [30] proposed a two-stage architecture based on a deep learning approach and a machine learning method. To improve color separation and contrast, Alkassar et al. [31] proposed a method that comprises normalizing the hematoxylin and eosin stains. Then, using two deep structure networks based on DenseNet and Xception, two categories of novel features—deep and shallow features—are recovered. To get the optimum performance, a multiclassifier method based on the maximum value is used.

To refine the network using histopathological images, Boumaraf et al. [32] applied the deep neural network ResNet-18 to breast histology images. The block-wise fine-tuning procedure is the foundation of the transfer learning approach. For the binary classification of breast pathological images, Zerouaoui and Idri [33] created and assessed twenty-eight hybrid architectures combining seven current deep learning techniques for feature extraction (DenseNet 201, Inception V3, Inception ResNet V2, MobileNet V2, ResNet 50, VGG16, and VGG19) and four classifiers (MLP, SVM, DT, and KNN). Liu et al. [34] proposed a breast cancer histopathological image classification method. They utilized ResNet-18, SENet, and maximum mean difference in their models to classify images.

In conclusion, although deep learning methods show significant improvements in detection and classification accuracies, the reduction of false-positive is still a challenge in breast cancer diagnosis. The motivation is to clarify the ability of the deep learning method to classify histopathological breast cancer. In addition, the histopathological image would help improve the sensitivity and specificity of the CAD systems. The proposed method can be summarized as described in Section 2.  Section 3 discusses the obtained results while the ablation study is discussed in Section 4. The conclusions of the study are presented in Section 5.

## 2. Proposed Method

The histopathology breast images required an automatic classification system because of the complex nature of these images. Histopathology images are colored images that make the traditional techniques inappropriate for successful classification. Histopathology images are in the RGB color space. So, color information is essential to distinguish between different types of carcinomas. We built a deep learning model to classify the histopathology breast images automatically. Discriminative features are primarily essential for successful classification. So, we proposed a residual deep learning model called the Histopathology-Net Model for robust computational systems.

### 2.1. Histopathology-RDNet Model

There are many challenges when increasing layers in a convolutional neural network (CNN) and going deeper. One of these challenges is the gradient vanishing problem, even if the network parameters are initialized carefully. Based on previous research, increasing the depth of the network will not address the degradation and the accuracy would not be improved [35]. In addition to these challenges, a deeper network has many parameters that require many images during training to adapt these parameters for generalization. The Histopathology-RDNet Model was proposed to classify the histopathology of breast cancer. So, going deeper requires a massive amount of histopathology breast cancer images, which may not be available. We utilized the residual learning method to overcome these challenges [36].

The novel deep learning “Histopathology-RDNet” used the residual learning technique. Residual learning reformulates layers and improves the information flow by skipping the connection of the layer inputs to address the degradation. Deep residual learning is working as CNN but with a special form. The weights of each layer are computed during training by the current training example and the previous decisions made on earlier training data. The input of the hidden layer comes from its immediate predecessor (input) layer and the previous output layer. So, the weight of any layer is calculated based on the input and the output prediction from the previous term by the next equation.

(1) $$\begin{array}{c} W_{s}x=F\left(W_{x},x\right)=F\left(W_{x-1}\right)\text{.} \end{array}$$

The proposed Histopathology-RDNet consists of 64 layers divided into a stacked-layer and a shortcut connection. The proposed model contains an input layer restricted to width (*W*), height (*H*), and depth (*D*), where *D* refers to the number of channels (red, green, and blue). In the proposed model, the input layer is restricted to *W* × *H* × *D* equals 300 × 300 × 3; several convolutional layers follow the input layer, batch normalization, pooling layers, dropout layer, rectified linear unit (ReLU) as activation layers, fully connected, and SoftMax layers.

Neurons in the convolutional layer are used to connect the subregions of the image. Image by image, the convolutional layer will learn to localize the features of image regions. The convolutional layers record the position of the features through the input images. Low-level features are learned in the first convolutional layers, while the higher-order features such as objects, shapes, and colors are learned in the deeper layers. After convolutional layers, a downsampling technique is required because the region of interest is not fixed. So, any slight movement in the input image produced a new feature map. So, a pooling layer is required to overcome this problem.

**Figure 1:**
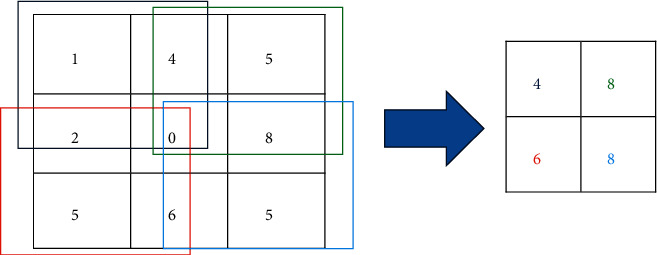
Max-pooling layer process [12].

We utilized max-pooling layers to downsampling the features map to extract the most significant elements in the input image. It returns the maximum value in the feature window map.  Figure 1 is plotted to show the output of the max-pooling layer. Each layer's parameters are updated to obtain the best performance during the training process. So, the stochastic gradient descent (SGD) updates the network parameters. SGD can address different issues. SGD follows the negative gradient of any objective when a few or only a single training example is fed to the layer. So, SGD updates the network parameters during training to obtain the best parameters setting. The SGD optimizer perceives excellent performance using a lower learning rate. So, the learning rate is updated to a lower rate every training epoch.

One of the main problems in the deep neural network is the difference between batch size (features map) from one layer to another. We utilized the batch normalization layer in the proposed model to overcome this problem. The batch normalization layer eliminates the internal covariate shift's effects by making the mean and variance standardization. The batch normalization layer is located after the convolution layer and before the RELU layer to reduce the problem of update coordination between layers.

**Figure 2:**
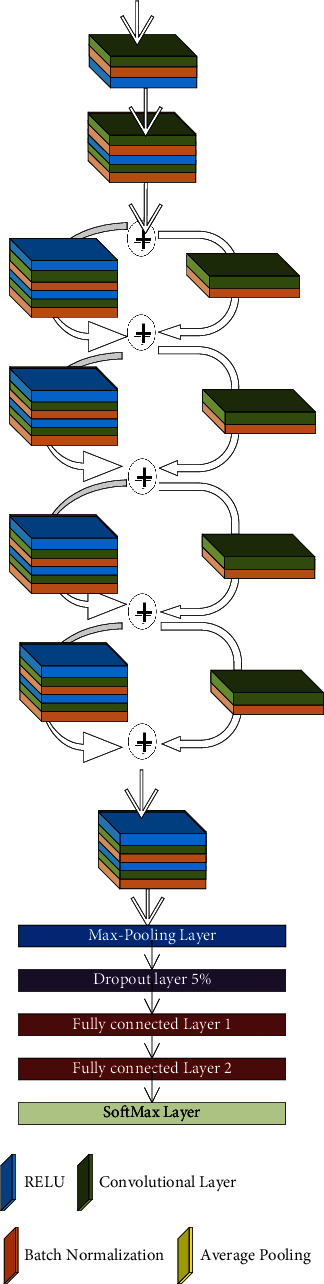
The proposed deep network.

RELU is a nonlinear activation function [37]. We utilized RELU followed the batch normalization layer because of gradient vanishing, sparse representation, and computational simplicity. It works by the thresholding method; all elements less than 0 are set to 0; otherwise, *x*. Finally, a fully connected layer transforms the input into an *N*-dimensional vector. Instead of using a sigmoid, a SoftMax layer followed the fully connected layer. Instead of the probabilities, summation must be 1 in the sigmoid; in the SoftMax layer, it may be 1. The value of the target class in SoftMax will have a higher probability.  Figure 2 shows the proposed model architecture.

## 3. Experimental Results and Analysis

### 3.1. Dataset

**Figure 3:**
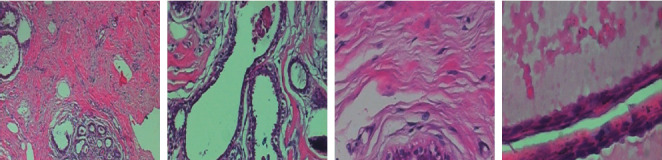
Sample images.

The BreakHis [38] database is the most widely used database for histopathological diagnosis of breast cancer among researchers. The BreakHis database includes 7909 images from 82 patients, which are arranged into four magnification factors: 40*X*, 100*X*, 200*X*, and 400*X*. Pathologists begin by recognizing ROIs in the lowest magnification level slide (*X*40), then go further into the latter using increasing magnification levels (*X*100, *X*200), until they have a profound insight (*X*400). To demonstrate this procedure, a BreakHis slide sample was collected with four different magnification levels. We randomly selected four images displayed in Figure 3. Each image is assigned to one of two categories: benign or malignant, with 2480 benign and 5429 malignant tumor images. Based on the appearance of the tumor under the microscope, each benign class is divided into four subcategories: Adenosis (A), Fibroadenoma (F), Phyllodes Tumor (PT), and Tubular Adenoma (TA). Malignant ones include Ductal Carcinoma (DC), Lobular Carcinoma (LC), Mucinous Carcinoma (MC), and Papillary Carcinoma (PC). A summary of image and patient distributions over main classes and different subcategories is presented in Table 1.

### 3.2. The Proposed Model's Implementation and Performance Metrics

**Figure 4:**
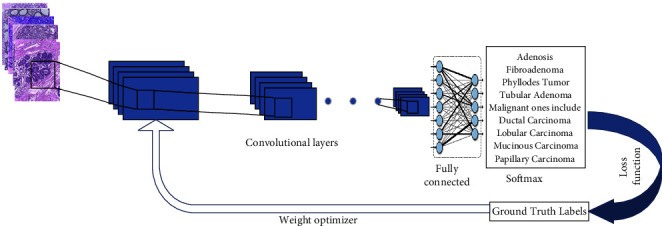
The overall process of the proposed method.

The proposed method has been coded and implemented over GPU using MATLAB 2018b x 64-bit. The studies were carried out on an IBM-compatible machine with a Core i7 processor, 16 GB of DDRAM, and an NVIDIA MX150 GPU. For all experiments, the batch size, maximum epochs, momentum, starting learning rate, and weight decay were set to 4, 50, 0.9, 0.00001, and 0.00001, respectively. The SGD is utilized to update the network parameters.  Figure 4 depicts the overall process of the proposed method. The proposed model has been evaluated using quantitative and qualitative measures. As a quantitative metric, the average measure of accuracy, sensitivity, specificity, and precision is computed using the following formulae. On the other hand, a receiver operating characteristic (ROC) is shown as a qualitative measure in the following equation:

(2) $$\begin{array}{c} Accuracy=\frac{t_{p}+t_{n}}{t_{p}+f_{p}+f_{n}+t_{n}}\text{,}\\ Sensitivity=\frac{t_{p}}{t_{p}+f_{n}}\text{,}\\ Specificity=\frac{t_{n}}{f_{p}+t_{n}}\text{,}\\ Precision=\frac{t_{p}}{t_{p}+f_{p}}\text{,} \end{array}$$

where *t*_*p*_, *t*_*n*_*f*_*p*_*f*_*n*_ refer to true positive, real negative, false-positive, and false-negative.

### 3.3. Results and Discussions

**Figure 5:**
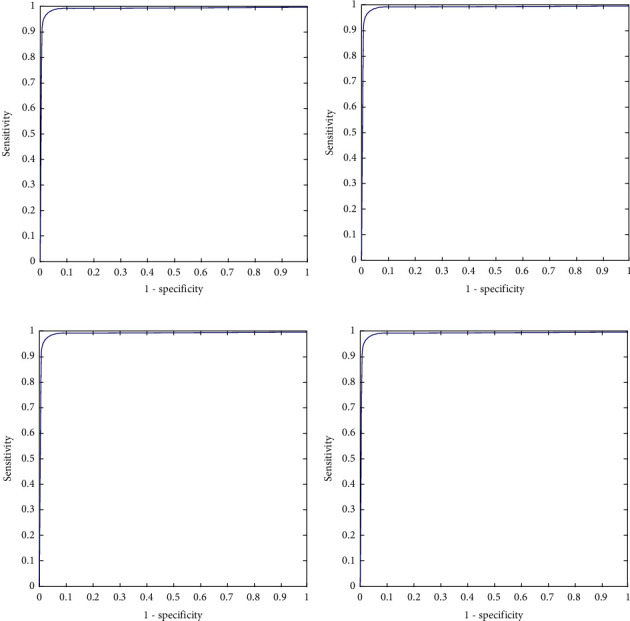
ROC curve of the classification results of BreakHis dataset using different magnification ratios. (a) ROC curve of magnification 40x. (b) ROC curve of magnification 100x. (c) ROC curve of magnification 200x. (d) ROC curve of magnification 400x.

The confusion matrix is applied to the test data to see the performance of the designed model. We evaluated the proposed model with different magnifying factors (including 40x, 100x, 200x, and 400x). The proposed model has been applied to classify the dataset into eight classes. Tables 2–5 illustrate the confusion matrix of the classification results of the BreakHis dataset using all magnifications of images.  Figure 5 shows the ROC curve of the proposed model over the dataset images in all magnifications.

The performance of the proposed model is shown in the confusion matrices Tables 2–5 and the performance measures shown in Table 6. It can be noticed that these matrices vary across different magnifying factors (including 40x, 100x, 200x, and 400x) using the same network parameters and the same number of test samples. The superiority of the proposed deep learning model is also shown in the ROC curve in Figure 5. It illustrates the ROC curve of applying the proposed curve of the classification results of BreakHis dataset using different magnifying factors (including 40x, 100x, 200x, and 400x). The proposed method was trained and evaluated using 8 more challenging classes than the state-of-the-art. According to the author's search, no study has beenpublished yet using the 8-classes for training and testing.

**Figure 6:**
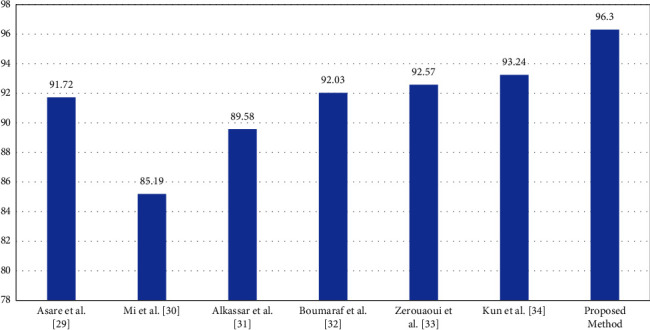
Results visualization for different methods.

From the obtained results, the proposed method can classify up to 8-classes with a classification rate higher than the other proposed methods in the literature review. Now, the proposed method will be compared with several methods [29–34]. The BreakHis dataset was used to analyze all of the state-of-the-art techniques to provide a fair comparison and evaluation with the strategy suggested in this paper. All the proposed methods in the previous work used deep learning with convolutional layers and transfer learning.  Table 7 shows and summarize the proposed methods while Figure 6 visualized the obtained results for methods [29–34] in addition to the proposed method.

The proposed method achieved high-performance measures that prove the proposed method's ability. These high measures were achieved because of several reasons. A full and robust automated breast cancer classification and high classification rates using residual learning with a new end-to-end trained deep neural network. Several convolution filters are applied to the same input in the suggested method. The proposed method uses different filter sizes that make the deep residual network extract feature accurate without using any preprocessing step such as segmentation or noise removal. The proposed model used skip connection to overcome the problem of overfitting. Several variables from several filters are combined to improve the effectiveness of breast cancer classification. The proposed method is able to classify multiclass instead of binary classification. We used cross-channel correlation instead of performing convolution on both spatial and channel-wise domain. The suggested model, unlike shallow networks, does not create substantial training mistakes. Another reason that proves the superiority of the proposed method is that no preprocessing step or augmentation has been carried out in the images before training and testing. On the other hand, the proposed method contains many layers with different convolutional filter sizes with a skip connection which is computationally expensive. The depth of the network is huge which requires a large number of images to adapt the appropriate parameters for the network.

## 4. Ablation Study

A model or algorithm's “feature” may be removed as part of ablation research to examine how performance is impacted. Understanding the impact of each of these breakthroughs independently is helpful. The easier it is to understand, the better (inductive prior to simpler model classes). Choose the less complicated alternative if two models perform equally but one is more complex. So, the ablation study can be done by comparing the proposed method with a simple and complex deep neural network using the same dataset. According to the literature, we found that Mi et al. [30] utilized inception V3. Inception V3 consisted of 48 layers while our proposed method consisted of 56 layers, which makes inception V3 simpler than the proposed method. But, the accuracy of this model is lower than the proposed method at 85.19% versus 96.3% on the same dataset and 8-classes. Another simple method using ResNet-18 was proposed by Boumaraf et al. [32], and Liu et al. [34]. These models obtain a lower accuracy rate equal to 92.03% and 93.24%, respectively, while the proposed model obtain 96.3%.

Asare et al. [29] and Zerouaoui and Idri [33] used much more complex models such as Inception_ResNetV2 and DenseNet 201 which consist of 164 and 201 layers, respectively. These models contain many more layers than the proposed model. The performance of these models was lower than the proposed method equal to 91.72% and 92.57%, respectively, while the proposed method obtained 96.3%. Finally, we investigated the effects of adding and removing a number of layers, and these impact the classification accuracy rate. As noticed, we find that the overall classification performance generally decreases as a result of ablations.

## 5. Conclusion

Compared to other medical imaging, histopathological images are the gold standard for diagnosing and categorizing breast cancer. Early detection is crucial in determining the best treatment plan for breast cancer. The primary motivation for developing a better breast cancer detection algorithm is to assist doctors familiar with the molecular subtypes of breast cancer in controlling tumor cell metastasis early in the disease's prognosis and treatment planning. So, a novel end-to-end deep learning model consists of different layers. The proposed method utilized the skip connection to achieve residual learning. The proposed method is able to classify up to 8-classes of histopathological breast cancer types. According to the different layers, filters, and filter sizes, the proposed method obtains higher performance measures compared with the literature. The proposed algorithm performs well in classifying complex-natured histopathological images of breast cancer.

## Figures and Tables

**Table 1:** The distribution of BreakHis images into four magnification levels for both main tumor categories and each subcategory.

Main category	Magnification level	Benign				Total benign	Malignant				Total malignant	Total of both
Subcategory		A	F	TA	PT		DC	LC	MC	PC		
Number of images at each magnification level	X40	114	253	109	149	625	864	156	205	145	1370	1995
	X100	113	260	121	150	644	903	170	222	142	1437	2081
	X200	111	264	108	140	623	896	163	196	135	1390	2013
	X400	106	237	115	130	588	788	137	169	138	1232	1820
Total		444	1014	453	569	2480	3451	626	792	560	5429	7909

**Table 2:** The confusion matrix of the classification results of BreakHis dataset using magnification 40x images.

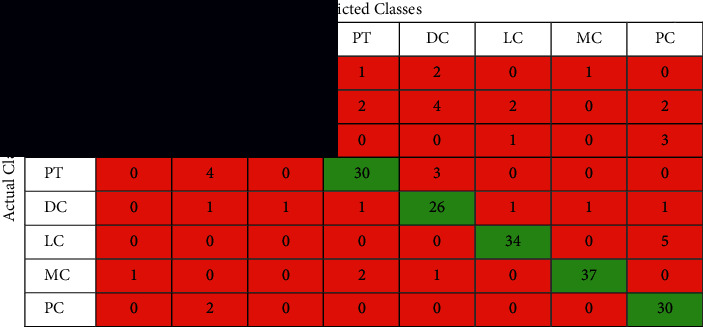

**Table 3:** The confusion matrix of the classification results of BreakHis dataset using magnification 100x images.

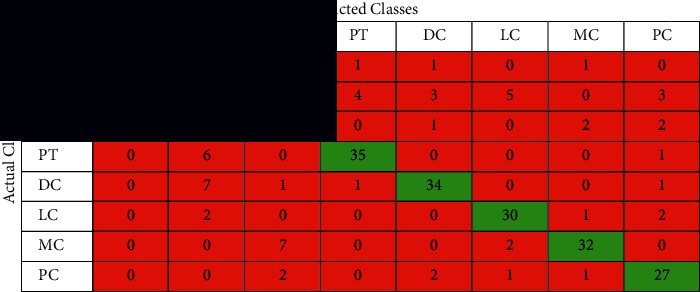

**Table 4:** The confusion matrix of the classification results of BreakHis dataset using magnification 200x images.

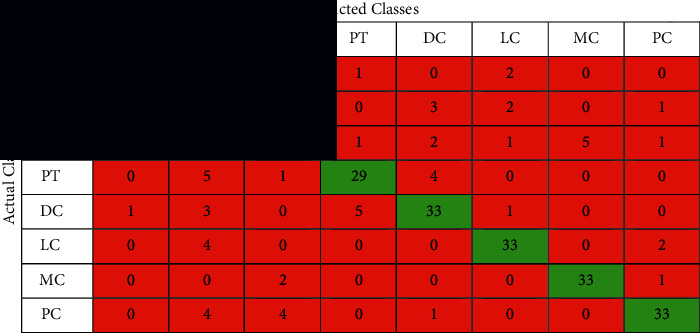

**Table 5:** The confusion matrix of the classification results of BreakHis dataset using magnification 400x images.

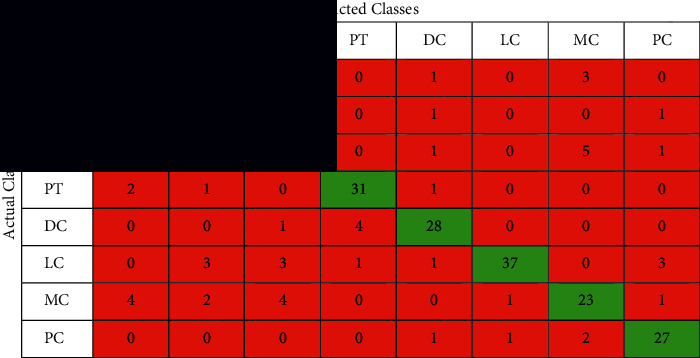

**Table 6:** The obtained result of the proposed method for classifying the BreakHis dataset for all magnifying factors.

	Average (%)			
	40x	100x	200x	400x
Precision	85.2	80	79.9	81.6
Sensitivity	85.2	79.1	79.9	80.7
Specificity	97.9	97.1	97.1	97.4
Accuracy	96.3	95	95	95.4

**Table 7:** The obtained result of the proposed method for classifying the BreakHis dataset for all magnifying factors.

	Method	Accuracy %
Asare et al. [29]	Inception_ResNetV2 with a Softmax as a classifier	91.72
Mi et al. [30]	Inception V3 with a Softmax as a classifier	85.19
Alkassar et al. [31]	Inception network with an ECmax as a classifier	89.58
Boumaraf et al. [32]	ResNet-18 with a Softmax as a classifier	92.03
Zerouaoui and Idri [33]	DenseNet 201 with a MLP as a classifier	92.57
Liu et al. [34]	ResNet-18 with a Softmax as a classifier	93.24
**Proposed method**	**Residual deep learning with a Softmax as a classifier**	**96.3**

## Data Availability

Breast Cancer Histopathological Database (BreakHis) data were used to support this study and are available at [https://web.inf.ufpr.br/vri/databases/breast-cancer-histopathological-database-breakhis/].
